# Applying Value Stream Mapping to Improve the Delivery of Patient Care in the Oncology Day Hospital

**DOI:** 10.3390/ijerph19074265

**Published:** 2022-04-02

**Authors:** Pilar I. Vidal-Carreras, Julio J. Garcia-Sabater, Juan A. Marin-Garcia

**Affiliations:** ROGLE, Departamento de Organización de Empresas, Universitat Politècnica de València, Camino de Vera s/n, 46021 Valencia, Spain; jugarsa@omp.upv.es (J.J.G.-S.); jamarin@omp.upv.es (J.A.M.-G.)

**Keywords:** healthcare operations, value stream mapping, lean healthcare, applications in healthcare systems

## Abstract

Improving the delivery of patient care is an ongoing challenge in the National Health Service (NHS). This challenge is not insignificant in the process of chemotherapy administration for oncology patients. The present research is motivated by a public Spanish hospital in which oncology patients receive medical care in the Oncology Day Hospital (ODH). At the ODH, oncology patients receive different health services by different specialists on a single day. Any discoordination in patient flow will contribute to longer waiting times and stays in the ODH. As oncology patients tend to have special health conditions, any extra time in the hospital is a source of risk and discomfort. This study applies value stream mapping methodology in a Spanish ODH to improve this situation, reducing hospital waiting times and shorting the length of stay. For that purpose, the path of the oncology patients is mapped and the current state of the system is analyzed. Working at takt time and levelling the workload are proposed for improving the working conditions for healthcare personnel. As a result, the quality of service for oncology patients who need a well-defined care profile is improved. The singular characteristics of the Spanish NHS make it challenging to implement new ways of working, so this study has significant theoretical and managerial implications offering directions in which improvement is possible.

## 1. Introduction

Cancer, as other important diseases, produces a major impact on people’s lifestyles and priorities. Nonessential matters are often pushed into the background while more vital matters, such as personal well-being and being surrounded by loved ones, come to the fore. According Dogan et al. [1], cancer is the second leading cause of death after cardiovascular diseases. To minimize the impact of cancer in patients’ lives [2] and as a result of substantial advancements in medical treatments [3], the Oncology Day Hospital (ODH) has come to be. The ODH [3] is also known as the Chemotherapy Day Unit (CDU) [4] or the Outpatient Chemotherapy Center (OCC) [5]. The distinguishing characteristic of the ODH is that it attends to patients as outpatients. This means patients do not need to spend the night in the hospital, unlike what happens with conventional hospitalization and traditional care [3]. The ODH’s distinctive approach has proven to be beneficial in reducing both the financial and psychosocial burdens for patients suffering from cancer [6,7].

In the ODH, oncology patients receive various health services in a single day [8]. The ODH health services are provided in several facilities and by different specialists, such as medical oncologists, nurses, and pharmacists [9]. It is worth remarking here that, depending on the chemotherapy treatment phase, oncology patients might be very weak and particularly sensitive. Therefore, careful coordination by the operations manager is required. Any discoordination in the flow will contribute to longer patient waiting times and stays in the ODH, which will ultimately translate into additional costs for the health system [10] and unnecessary discomfort for oncology patients [11]. In fact, according to the related literature, waiting times are the main source of dissatisfaction among oncology patients [2,4,12,13,14,15].

The need for coordination of the different services and resources associated with ODH and their complex interdependence is a problem addressed in the literature from different points of view. Lame’s study [16] reviews the contributions to operations management and operations research literature that address the planning of outpatient chemotherapy. This work describes in detail the organizational aspects of outpatient chemotherapy through the reviewed works, they being a good starting point to understanding the complexity of the problem. According to their study, only one paper of all the papers analyzed describes a hospital with a centralized pharmacy from a systemic perspective, encompassing both the pharmacy and the outpatient clinic [17]. However, as the author points out, the case of general hospitals with centralized pharmacies is a very common situation in European hospitals and more research is needed in this area. Some recent studies in scheduling chemotherapy appointments proposed optimization approaches and algorithms to improve the work procedures of the ODH, develop scheduling methods for resource utilization optimization, and decrease patient wait times for chemotherapy [8,11,18,19]. These approaches are very promising for optimizing already established processes. However, if the ODH is at a stage prior to the optimization models and requires improvement of the processes themselves, it is reasonable to use qualitative methods such as “lean” to reduce non-value-added processes, identify primary sources of inefficiencies, and reduce patient wait time [20].

Lean is one of the most common approaches to create and maintain flow [21,22,23]. The lean approach is an integration of concepts, methods, and tools whose main goal is to be extraordinarily customer-oriented and responsive by ridding the entire system of waste, thereby delivering to a customer exactly what (s)he wants, when (s)he wants it, defect-free and on time. Lean originated in the manufacturing industry [24], but its advantages have been leveraged by other sectors, such as the health sector [25,26,27,28,29,30].

The use of lean in the health sector is known as lean healthcare. Lean healthcare has recently seen numerous applications in healthcare institutions [31] such as hospitals [32,33], clinics [34,35], and medical centers [36,37]. Dahlgaard, Pettersen, and Dahlgaard-Park [38] describe lean healthcare as a management philosophy that develops a culture characterized by increased satisfaction for patients and other stakeholders through continuous improvements, in which all employees actively participate in identifying and reducing non-value-adding activities (i.e., waste or “*muda*”). Healthcare operates in a complex professional service environment that faces challenges in delivering high quality and affordable care—a set of goals that some healthcare professionals believe is incongruous [39]. This challenge highlights that the system and the managers have no room for non-value-added activities.

There are multiple tools and techniques to help managers to implement lean but value stream mapping (VSM) should be the starting point [40,41]. As exposed by Rother and Shook [42], VSM serves to map the flow of information and processes in a system. Mapping the flow allows the identification of the sources of waste and the proposal of improved future scenarios [43,44,45]. Related literature also recognizes that VSM is a powerful tool to minimize the time that patients spend in the hospital and to reduce traditional management costs [46,47].

Despite the growing number of empirical research articles [20,32,33,35,48] and literature reviews on lean healthcare [20,30,49,50,51,52,53,54], few applications are reported in the Spanish National Healthcare System (NHS) [55]. This may be explained by the singular characteristics of the Spanish NHS that differ from other European healthcare systems [55]. The Spanish NHS is supported by public funding and the healthcare staff is mainly composed of civil servants or workers dependent on the NHS [56]. These distinct features make it challenging to implement new ways of working, even when these new ways could be highly convenient for the patients and for the healthcare system itself.

Furthermore, there is even fewer applications of VSM to the Spanish NHS [57]. According to recent reviews [41,47,57], and after applying a robust and exhaustive search protocol [58], only two articles that describe and apply VSM methodology to the Spanish NHS were retrieved. The first article [59] applies VSM to the clean room of the pharmacy department, in a hospital of Las Palmas de Gran Canaria, to reduce non-value-adding steps. The second article [60] applies VSM to the intensive care unit (ICU) of a tertiary hospital to improve the flow and care of critically ill patients, and to increase professional satisfaction. These two articles improved the management of the hospitals but neither of them considered the ODH. In this paper, the researchers studied an ODH integrated into one of the public reference hospitals in Spain that attends to more than 300,000 patients per year. The goal of the study is to use VSM to improve the provision of health care to oncology patients and the working conditions for the healthcare personnel. The VSM methodology is used to design a future state map that minimizes the duration of non-value-adding activities, such as waiting times and patient stays.

The remainder of this paper is organized as follows. Section 2 presents the framework and the methodology for the project. Section 3 describes and analyzes the current state of the system. Section 4 proposes a set of changes to improve the way the ODH’s oncology patients are attended and defines improved future states of the system. Finally, the theoretical and managerial implications, as well as an outline of research limitations, are included in the Section 5.

## 2. Framework and Methodology

### 2.1. Framework: Health System Context and Oncology Day Hospital

#### 2.1.1. Health System Context

The Spanish NHS healthcare system follows the principles of the Beveridge model [61]. The NHS is protected by the Article 43 of the Spanish Constitution (1978), which establishes the right to healthcare protection for all Spanish citizens. Some of the features of the Spanish NHS include public funding, universal coverage, and unlimited healthcare services. The Spanish NHS is directed by the central government administration and coordinated with the Spanish Autonomous Communities, which are legally responsible to provide the healthcare functions and benefits.

As discussed before, the nature of the contracts held by the health professionals constitutes a very distinct characteristic of the Spanish NHS. Health professionals are salaried workers and a large proportion of them have a special civil servant status (i.e., “statutory staff”) [62]. As civil servants, they hold their job positions for life and their salary costs are financed from general taxes [63]. However, the Spanish NHS also adopts more precarious forms of contracts, such as part-time and day contracts, to cover shortages or emergency services. In addition, public employee pharmacists in both primary health care and hospitals are also salaried [62].

The basic salary for statutory staff is regulated by the national government, but regions have the capacity to vary some components that make up the total salary. One of the drawbacks of this payment system is that it is considered to be rigid and inefficient at rewarding the efficiency and quality of the work provided by healthcare professionals [62].

#### 2.1.2. Oncology Day Hospital (ODH)

The Oncology Day Hospital (ODH) studied in this paper is part of the Spanish NHS and it is integrated into a large public hospital. This hospital attends oncology patients in every stage of the disease: the diagnosis phase, the treatment phase, the discharge phase, and the follow-ups. The treatment phase, in which the different cancers are treated by chemotherapy, takes place at the ODH and is the one analyzed in this paper.

The ODH provides chemotherapy treatment to 28,000 patients. The ODH team is composed of oncologists and oncology nurses, as well as pharmacy and laboratory services. It is worth mentioning that the pharmacy and laboratory services are not exclusive for the ODH but are shared with the rest of the hospital services. The ODH is coordinated by a medical oncologist and an oncology nursing supervisor.

### 2.2. Methodology: Value Stream Mapping

Value stream mapping (VSM) is a pencil-and-paper tool to describe and understand the flow of material and information of a product moving through the value stream [42]. VSM is a simple tool to improve a process by making changes that are specific and sustainable over time [42]. Rother and Shook [42] list four steps to follow when applying VSM methodology to the clinic environment:

1. Identifying the “family of patients” to be mapped;
2. Drawing the current state map;
3. Drawing the future state map;
4. Designing an implementation plan to reach the future state.

The first step is crucial as it sets the scope of the project by establishing the flows to be improved and the flows to be ignored. The second step provides a critical and detailed analysis of the actual state of the system. The third step defines the future state and the tools that should be implemented to achieve it. Finally, the fourth step presents the implementation plan to achieve the future state.

For the development of this methodology to be adjusted to the Spanish ODH framework, it is necessary to start from these assumptions:

- The number of ODH human resources (medical oncologists, nurses, and pharmacists) and their salaries cannot be increased;
- No changes can be made to the facilities or material resources (beds, armchairs) of the ODH;
- Changes to ODH processes that affect the processes of the entire hospital require extensive time to be implemented;
- Changes to ODH processes that involve another medical institution will be directly managed by the hospital’s chief executive officer (CEO) because some changes must be accepted by the regional government.

According to the first step of the VSM methodology, the family of patients for this study will be oncology patients who attend the ODH integrated in the Spanish public hospital. As discussed before, these particularly sensitive patients usually complain about long waiting times at the hospital, so improvements are necessary and required. Note that pediatric oncology patients are not included in the family of patients because for these patients the hospital has a priority established that does not need substantial improvement.

The researchers created a multidisciplinary lean team to develop the methodology. The team included the medical staff involved in the patient pathway (i.e., oncologists, oncology nurses, pharmacy, and administrative personnel) and the experts in the lean methodology (i.e., the researchers). The team was led by the lean experts who developed and ran a workshop financially supported by the hospital [64] on basic lean principles and tools (e.g., 5s, KPI, standardization, VSM, etc.) to engage the team with the improvements.

The development of the rest of the methodology is included in the following sections. The second step is developed in Section 3, and the third and fourth steps are developed in Section 4. It is important to note that the specific plan for the implementation of the future state is not provided as such. The reason for this is that the future state will be directly managed by the hospital’s chief executive officer (CEO) and because some changes must be accepted by the regional government. Nevertheless, detailed information on how to implement the future state is included.

## 3. Current State Map and Analysis

Once the family of patients is selected, the next step for the team is to “go and see by yourself” (i.e., “*gechi genbutsu*”). This means go to the workplace and observe and understand the current situation. Although not following the direction recommended by Rother [37], the data were taken in the direction of the flow. This decision was made based on the researchers’ experience, which demonstrates that following the direction of the flow is easier in services areas with few trained people, as is the case in the present study. Specifically, the team followed the flow of the patients from the time they arrive at the hospital until the time they go home.

### 3.1. Current State Map

When oncology patients visit the ODH, they have three medical appointments scheduled on the same day. The first one is for blood collection and occurs first thing in the morning. The second one is to see their oncologist and occurs at least 1 h after the blood collection. The third one is for chemotherapy administration and occurs at least 3 h after the oncology appointment.

The ODH studied in this paper is divided between two floors of the hospital. The first floor has an area with armchairs and beds where blood is drawn and chemotherapy treatments are given. These services are provided by the oncology nurses. The second floor includes the oncology consultancies area and is shared with outpatient consultations. These oncology consultancies are provided by the oncologist. In the ODH, waiting rooms per se do not exist and only some benches are provided. Normally, these benches are placed around the room for drawing blood and chemotherapy administration or around the consultation rooms. Although this design is common in Spanish public hospitals, it represents a major drawback for oncology patients because it exacerbates problems associated with the administration of chemotherapy, such as the risk of catching diseases and general weakness and discomfort.

The detailed description of the flow for ODH patients is described below:

1. Patients arrive at the hospital and check in at the self-service kiosk.
2. Patients wait to be called by the nursing staff.
3. Patients enter the ODH to undergo blood tests. Oncology nurses are responsible for drawing and delivering blood samples to the laboratory.
4. Patients go to the second floor and check in via the kiosk. Patients wait for at least 1 h before being attended by the oncologist. In the meantime, the blood samples are tested in the laboratory and the results are uploaded to the computer application.
5. Patients are attended by the oncologist. Based on the results of the blood samples, the oncologist adjusts the chemotherapy treatment for the patient. This information is received by the pharmacy department, which will then prepare the medication.
6. Patients return to the first level and once again they check in via the kiosk. Patients wait for more than 3 h until they start receiving chemotherapy.
7. Patients enter the ODH to undergo chemotherapy. The oncology nurses are responsible for administering chemotherapy. Chemotherapy treatments may last between 15 min and 4 h, depending on the type of cancer.
8. Patients go home until their next medical check-up.

**Figure 1:**
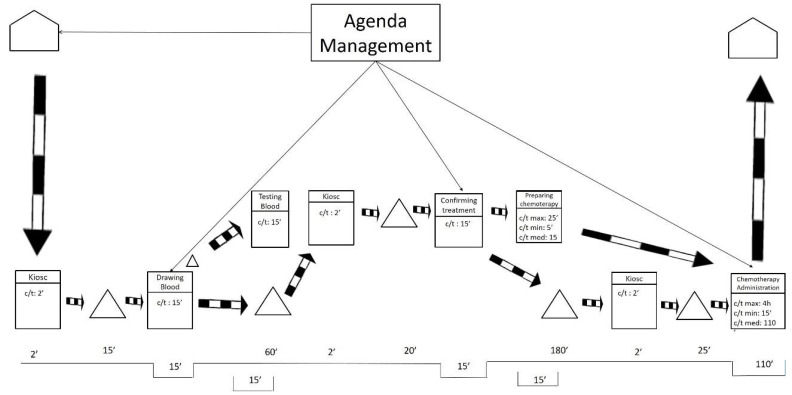
Current state map.

After visiting the workplace and collecting the data, the team represented the current state map using the post-its technique. As shown in Figure 1, the researchers also drew the current state map in a digital format to facilitate communication with other hospital members.

As displayed in Figure 1, the current state map is divided into two levels. These levels mirror the floors of the ODH. The lower level includes blood extractions and chemotherapy treatments. The higher level includes oncologist consultations.

On the one hand, Figure 1 exposes how the processes of preparation and administration of chemotherapy have variable durations (max., min., and medium). For these processes, the average duration is weighted based on the number of patients of the different types of cancer and the duration of their respective treatment. On the other hand, the duration for taking blood samples, testing the samples, and medical consultations is more stable, so the average duration is directly calculated.

The current state map only represents information that is directly related to the flow of patients at the ODH. That treatment agenda was completed by the agenda management. This process was as follows: once patients are diagnosed and their treatment is determined, an attempt is made to adjust the treatment appointments required to fill any spaces in the agenda of the medical oncologist who does the follow-ups, without bearing in mind the workloads involved for each patient during each particular session held at the ODH. Apart from these information flows, we found the typical flows that result from saving reports, test results, are immediately saved and do not affect the patients’ flow, nor the quality of care, at all.

While monitoring the flow of the patients, the researchers also interviewed the staff involved in the processes. The most remarkable comments are listed below:

1. Patients have to wait to be attended despite having three scheduled appointments. These waiting times are often extended because of delays in the process.
2. Patients often complain about waiting long hours at the ODH. In some cases, patients spend even more than 8 h in the ODH.
3. Oncology nurses work in fits and starts. In the mornings, nurses experience higher workloads because all the patients arrive at the same time for blood collections. Then they have a “resting” period with very little work to do. Finally, patients come back at irregular intervals to be administered chemotherapy treatment.
4. The job of the oncology nurses does not just involve blood specimen collection and treatment administration but they also attend to patients individually by answering their questions and by offering them human support. Oncology nurses reported that rush hour is the most complicated time to fulfill all these tasks because they need to administer treatment to too many patients. At other times, however, oncology nurses declared they have plenty of spare time. This tends to be the case on Fridays, when the type of chemotherapy treatments provided take longer and there is no free space to accommodate more patients. At these times, the seats for blood collection and chemotherapy administration are collapsed and become a limiting resource.
5. Oncology nurses also stated that their workloads were systematically unbalanced. Indeed, they reported that the days on which their workloads are heavy tend to happen during the same weekdays.

### 3.2. Metrics

Table 1 presents the main metrics and the most adopted metrics of the current state map [65]. It can be observed that some metrics are affected by the variable duration of the chemotherapy preparation and administration. These metrics are value-added time, length of stay, and value-added rate. The duration for other metrics, such as the patient’s wait time and non-value-added time, remain consistent regardless of the duration of chemotherapy.

### 3.3. Analysis of the Current State

**Figure 2:**
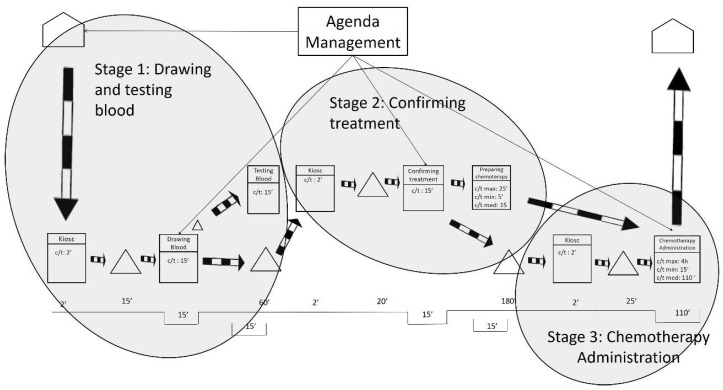
Stages in the current state map.

After gathering the quantitative and qualitative data and representing the current state map, the results were analyzed. As shown in Figure 2, the researchers defined three stages based on the fixed waiting times between activities. These fixed waiting times are a result of the lack of flow synchronization in the system. It is worth mentioning that waiting times may be increased by some variables such as unpunctual patient arrivals or difficulty in finding a patient’s vein when extracting blood samples. The three stages are related by information flows as patients have appointments at a given time to take the blood samples, to be seen by their oncologist, or to be administered the chemotherapy treatments.

The first waiting time occurs after the blood samples are drawn, and it sets the end of Stage 1. The first waiting time is the time the laboratory spends testing the blood samples from the oncology patients. Interestingly, the laboratory could test the samples in less than 15 min. However, oncology patients need to wait 1 h for their next appointment to ensure that the test results are available for consultations. This translates into longer waiting times for the patients and is sometimes compounded by the fact that 1 h is not even enough time to obtain the results. As indicated by the hospital staff, delays often occur because the laboratory receives all of the hospital’s blood samples at the same time—normally first thing in the morning, between 8:30 and 10:30 a.m. In addition, all the blood samples are received with no priority, which makes the laboratory process collapse.

The second waiting time is the longest and occurs after the patient visits the oncologist. This is the end of Stage 2 and is the time the pharmacy department spends preparing the treatments requested by the oncologist. The pharmacy department claims it cannot guarantee supplying medication in less than 3 h after receiving the order. The researchers analyzed the treatment preparation process and observed that dose preparations take only 15 min on average. Some medications can be prepared in 5 min, and others take up to 25 min. The analysis unveiled that the causes of delay in the pharmacy were like those in the laboratory: all the hospital medication requests arrive at the pharmacy department at the same time and with no priority (except for pediatric oncology treatment). This means that preparing treatments for hospitalized patients (e.g., inpatients), who may not have scheduled times to take the medication, has the same priority as preparing medical treatments for oncology outpatients. This seems to be unbalanced because, as highlighted before, oncology patients tend to have low defenses and insufficient waiting area spaces. During the analysis, it was also observed that certain problems related to the distribution of the medication contributed to slowing down the process.

The length of the third waiting time is variable, and it is a direct consequence of the two previous waiting times. This is because the waiting times in Stage 1 and Stage 2 produce unequal workload periods for oncology nurses. As previously mentioned, all blood samples are taken first thing in the morning; however, oncology patients do not receive the chemotherapy treatment until 4 h later. During these 4 h, the oncology nurses do not have patients to attend to and use their time to review reports and to prepare and clean areas. After these 4 h, most of the oncology patients are ready to receive chemotherapy treatment. This implies that the oncology nurses need to attend again to a large number of patients almost at the same time. The nursing staff also declared that some days not all oncology patients receive the same treatment, nor require the same specialization of care from the oncology nurses. In summary, the peaks of patient flow often encounter a lack of human resources (e.g., oncology nurses), or material resources (e.g., beds, armchairs), or both.

**Figure 3:**
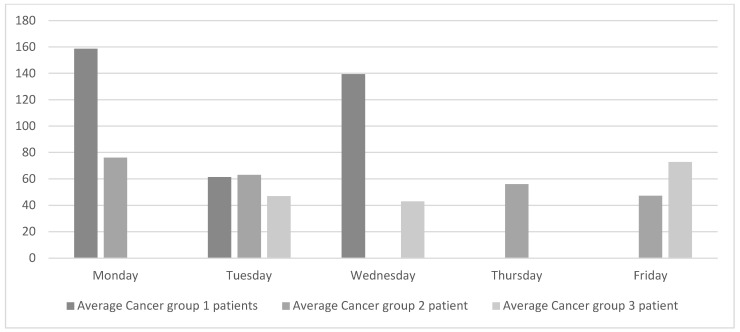
Current daily average of oncology patients seen by the medical oncologist.

The analysis of the current state was complemented by collecting data on oncology patients visiting the oncologist each day of the week. The data were classified into cancer groups as shown in Figure 3. Patients in group 1 are generally on short treatments and the chemotherapy treatments take between 15 and 40 min. The chemotherapy treatments for patients in group 2 have an average duration of 2.5 h, and the treatments for patients in group 3 are the longest and last between 3.5 and 4 h.

Figure 3 reveals that the flow of patients attended by the oncologists at the ODC is significantly unbalanced both within and between each cancer group. For example, patients in group 1 visit the oncologist on Mondays, Tuesdays, and Wednesdays. However, the number of patients on Tuesday is less than half the number of patients on either Monday or Wednesday.

Figure 3 also shows that Friday is one of the days with the least number of patients. However, oncology nurses confirmed that the waiting areas are still full of patients and, even then, some of them have to wait. This is because very long chemotherapy treatments tend to be administered on Fridays. On Mondays, however, oncology nurses reported that the waiting areas are emptier, although they still need to rush to be able to attend to all the patients.

It is important to remark that oncologists are specialized in a specific type of cancer and although they have consultations every day of the week, they only attend to oncology patients at the ODH 3 or 4 days per week. Oncologists spend the rest of the weekdays following other cancer-related processes, such as diagnoses and follow-ups. The researchers attempted to learn more details about this singular scheduling system, but no satisfactory answers were found.

In summary, the analysis of the current state shows that nurses’ workloads significantly fluctuate throughout the day. Heavy workloads occur in the morning (e.g., morning blood tests) and in the afternoon (e.g., chemotherapy treatments), while lighter workloads occur in the middle of the day. The analysis also exposed that the amount of work also varies significantly each day of the week. This is largely explained by the fact that each type of cancer requires a very different type of treatment.

## 4. Future State Map

This section proposes a set of changes to improve patient care and prevent fluctuation in the amount of the nurses’ work. It is important to emphasize that, in any future scenario, the construction of a specific waiting room for oncology patients is recommended. However, the researchers are aware that, despite being a large hospital, the facility is already deeply saturated because of its structural design.

The future map was developed applying two simple lean concepts: work at takt time and workload levelling (i.e., “Heijunka”). Working at takt time is defined as the ideal pace at which the health staff should work based on the amount of work required and the time available to perform it. Workload leveling aims to level out the workload and services provided by the ODH.

As explained in detail below, the researchers proposed to adjust the pace at which oncology nurses work throughout the day to meet the takt time. Working at takt time will contribute to levelling daily workloads. The researchers also proposed to level out the oncologists’ workloads throughout the week. By extension, levelling oncologist’s workloads will smooth out the weekly workloads for the oncology nurses.

- Oncology nurses: working in takt time and levelling the daily workloads to avoid peaks:

As mentioned before, the oncology nurses often work in fits and starts. They have a heavy amount of work first thing in the morning obtaining blood samples almost simultaneously from all the oncology patients. Then their workload lightens for a few hours until midday. After that, the amount of work peaks again when all the patients are ready to receive chemotherapy treatment.

To avoid having starts and stops, patients need to be attended at a stable and regular rate. The researchers proposed taking the blood samples the day before or distributing them throughout the day. This solution will allow oncology nurses to work at takt time and to smooth out their workload. The ODH healthcare team assessed these solutions and confirmed its feasibility. Since the blood collections do not necessarily need all the patients to have empty stomachs, they can happen at any time of the day. Moreover, the blood collections do not need to happen the same day as the oncology consultation, so taking them the day before is also a potential solution. These changes will also help the laboratory. For example, it will be able to prioritize the blood tests for patients who need to come in with empty stomachs. Let us remember that the ODH is integrated into a large hospital and that the pharmacy and laboratory services are shared with the entire hospital, so careful coordination is required.

**Figure 4:**
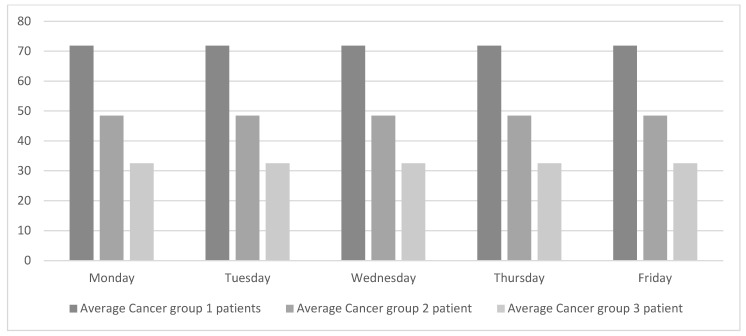
Proposed daily patients’ average per specialty.

Levelling the workload involves doing all sorts of tasks alternatively and throughout the day. To help the oncology nurses to achieve that, the following guidelines were proposed:

- Do not limit blood collections to first thing in the morning. Patients should be scheduled as evenly as possible throughout the entire day.
- Administer chemotherapy treatment by starting as soon as possible and continuing throughout the entire day. As for blood collections, lags between treatments should be as constant as possible. The input (e.g., patients starting chemotherapy treatments) to chemotherapy administration will be steady and in takt time, but the output (e.g., patients leaving the chemotherapy treatment) will not be as constant because of the different durations of the treatments for each type of cancer.

- Oncologists: levelling the weekly workloads to achieve a smooth workflow:

Levelling the weekly workload equally across all the weekdays will regulate the flow of patients and consequently improve patient-care quality. As shown in Figure 3, oncologists do not work with a single type of patient every day. Instead, different types of patients are grouped into lots. This way of working has been in place for many years, probably because it simplifies the ODH’s working schedules. Although this way of working suits the oncologists, it hurts the overall chain of value. The objective of this study is to achieve global improvement, and for that purpose the weekly workload is smoothed out, as shown in Figure 4. 

To achieve the workload profile presented in Figure 4, a tool will be needed. This tool will coordinate the ODH, which is exclusively dedicated to oncology patients, with the rest of the oncologist consultations. This tool should also be needed to adjust the workloads based on other variables (e.g., weeks with vacation days). The researchers were informed that the hospital is currently working with the oncologists to schedule treatment cycles.

### 4.1. Ideal Future State Map

Considering the lean principles proposed in the previous sections, and with the aim of minimizing patients’ waiting times, three future states are proposed. One of the future states is ideal and will be difficult to apply; the other two are feasible solutions that would be relatively easy to implement. First, the future states will be described and then the results for the three future states will be compared to the current state.

**Figure 5:**
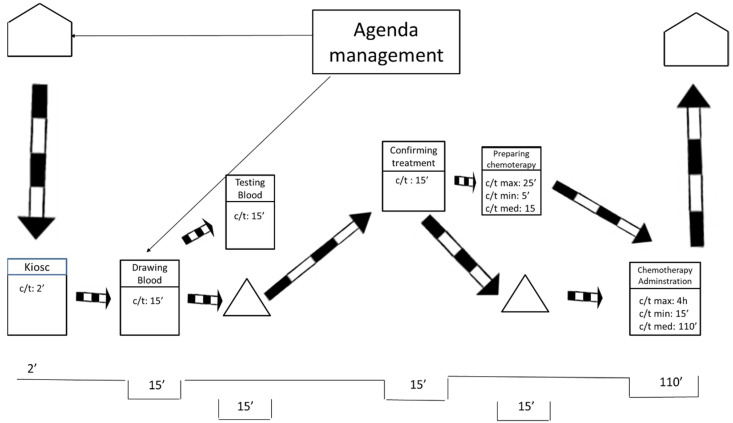
Ideal future state map.

Pursuing continuous flow is the basic guideline for creating the ideal future state, along with working at takt time and levelling the workload. According to these premises, the ideal future state map is shown in Figure 5.

Figure 5 portrays an ideal situation in which only one flow of information exists. The flow of information occurs at the beginning of the process and the rest operates by push. In this ideal future state, the average waiting time is reduced to 30 min. That is a vast reduction from the original waiting time of 300 min. The ideal waiting time of 30 min is composed of the time the laboratory needs to obtain the blood tests results and the time that the pharmacy needs to prepare the treatment.

To make this ideal future state possible, it would be necessary to level out all the workloads in the entire hospital—not only in the ODH but also in all the hospital departments that send blood samples to the laboratory and in all the sections that require the hospital’s pharmacy department. These conditions make the ideal future state unfeasible, at least in a reasonable time, as they imply a global change in a hospital that employs thousands of workers.

### 4.2. Feasible Future States Maps

After considering that changing the way the entire hospital works is only possible in an ideal future, two feasible future state maps with partial improvements are presented. As before, the objective of these future states is to minimize patient waiting times at the ODH by working at takt time and levelling the workloads. The authors are aware that these two more realistic scenarios do not fully achieve the basic lean principle of creating flow. However, they achieve the ultimate end, which is improved patient care.

#### 4.2.1. Feasible Future State 1

**Figure 6:**
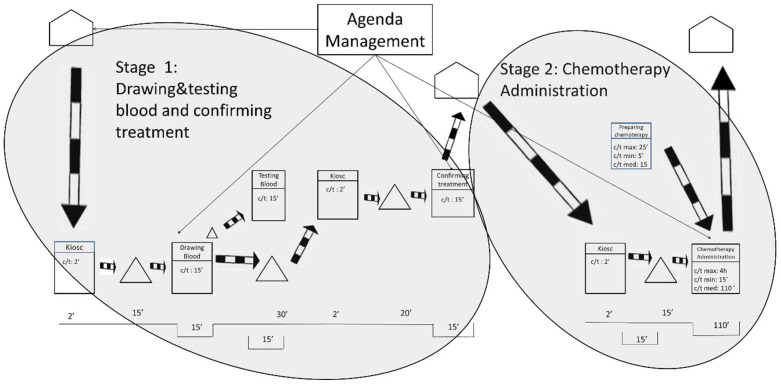
Future State Map 1.

The future state 1 was designed for those patients who live in the city and its surroundings, that is, approximately a 1-hour roundtrip by car or public transportation. As shown in Figure 6, the researchers propose to split the process into two stages that will occur in 2 consecutive days. The 1st day patients will arrive at the hospital at the time of their appointment to undergo the blood tests and to visit the oncologist. The 2nd day patients will visit the hospital only to receive the chemotherapy treatment.

Splitting the process in two stages will allow the laboratory to prepare the treatments in advance and will benefit the oncology patients by reducing the longest waiting time between the oncology consultation and the chemotherapy treatment. The goal is to ensure that the maximum waiting time is never greater than 15 min.

Appointments on both days should be conveniently distributed throughout the day to avoid workload peaks and favor working at takt time. The future state 1 would allow oncology nurses’ workloads to be leveled throughout the working day. This is utterly important because smoother workloads for the nursing staff are associated with improved patient care [66]. The future state 1 will also contribute to improving the conditions for the laboratory by allowing it to prepare chemotherapy treatments before the patients visit the ODH. Moreover, oncologists will also be able to better level their workloads, which will undoubtedly contribute to shorter waiting times for the oncology patients.

The obvious drawback of this solution is that patients need to go to the hospital for 2 days instead of 1. However, patients were interviewed and 90 per cent of them reported that they would prefer this future state over the current situation because waiting times are still reduced. From the ODH management’s point of view, the implementation of this future state is simple and will only require an internal change in the agenda management.

#### 4.2.2. Feasible Future State 2

**Figure 7:**
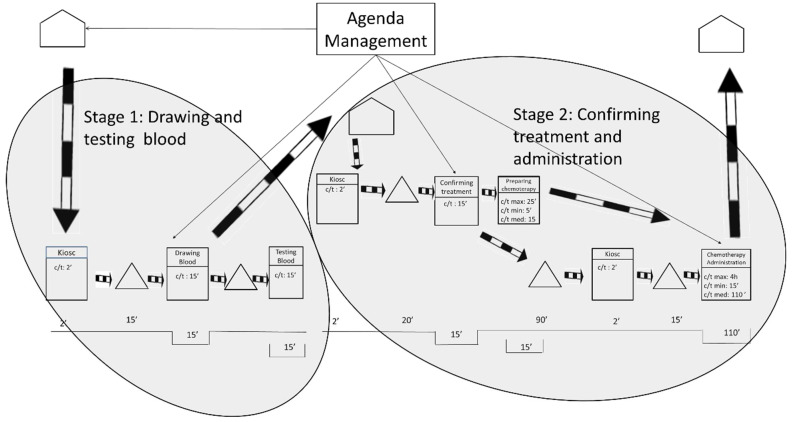
Future State Map 2.

The future state 2 was designed for patients who live far away from the hospital. It is understood that for these patients it will be an inconvenience to travel to the hospital twice. As shown in Figure 7, the researchers propose again to split the process into two stages. As before, phases occur on 2 consecutive days but this time patients will visit a different health institution on each day. The 1st day patients will visit their nearest medical center and on the 2nd day they will visit the ODH.

The 1st day patients will visit their nearest medical center only for blood collection, and they will not have to wait for the results to be ready. Spanish medical centers schedule blood draws first thing in the morning. The time the blood is waiting to be tested is variable and will depend on the organization of each medical center, but all the test results will be available to the ODH by the morning of the next day. The 2nd day patients will visit the ODH, see their oncologist and undergo the chemotherapy treatment.

Since all the stages are not carried out in the same health institution, a cost redistribution will be required among the health institutions. This cost redistribution will be managed by the hospital’s chief executive officer and agreed to by the regional government. As with future state 1, these improvements involve two visits, but the patients still report that they will prefer this future state because waiting times are reduced.

### 4.3. Benefits for the Future States Maps

Table 2 summarizes the values for the measured metrics in the current state and in the proposed future states. Table 2 clearly shows substantial improvements for all the metrics in the ideal state but, unfortunately, this state is not viable for the hospital at this moment. The overall improvements for the future states are summarized herein:

- Waiting times are reduced in all three future scenarios. As compared with the current state, the waiting time is reduced by 90 per cent in the ideal state, by 73 per cent in future state 1, and by 25 per cent in future state 2.
- Non-added-value activity times are also consistently reduced for all three future scenarios. This is a direct consequence of the reduction on the waiting times. As compared with the current state, non-value-added times are reduced by 89 per cent in the ideal state, by 71 per cent in future state 1, and by 49 per cent in future state 2.
- The length of stay is also reduced in the three future scenarios. This is a direct consequence of reducing waiting times and non-value-added activities.
- The value-added rate (VAR) is increased. This is expected since VAR is the ratio between valued-added time and the length of stay.

By reducing the waiting times, the study achieved the reduction of the main source of dissatisfaction for oncology patients. Moreover, levelling the workload and working at takt time contributed to achieving better work conditions for the health providers. These improvements will have a positive influence for oncology patients as they are closely related to every aspect of patient experience, including confidence in the care provider and perceived quality of care [66]. We should not forget that a heavy nursing workload seems to be related to suboptimal patient care and may lead to reduced patient satisfaction [67].

The future states will benefit all the health care involved with the ODH. Oncology nurses reported that they were less stressed and able to provide better service to the patients. The laboratory and pharmacy department will be able to better distribute their workloads and avoid the peaks. For the oncologists, this new way of scheduling is an opportunity to improve how they manage patient care. All in all, it can be stated that these benefits achieved improvements in how oncology patient care is provided.

## 5. Conclusions

This paper applied the VSM methodology to an oncology day hospital (ODH) integrated into the Spanish NHS. The VSM mapped the oncology patients who attend the ODH to receive chemotherapy treatment. The value stream maps were used not only for diagnosing the situation, but also as a communication tool between hospital managers and the medical staff. The analysis of the current state of the site unveiled that sensitive oncology patients were unsatisfied because of long waiting times, that oncology nurses were experiencing high workloads, and that the laboratories and pharmacies were overburdened. The analysis also shows that patients visit the oncologist in lots, according to their cancer type. Using the lean approach, the authors proposed changes to resolve these frictions and improve how patient care is provided. Fundamental lean concepts such as working at takt time and workload levelling were applied. Three future states of the site, which reduced oncology patient waiting times and non-value-added activities time, were proposed through three different value stream maps. The benefits of the future states were reported using common metrics: wait time, non-value-added activities times, value-added activities times, length of stay, and value-added rate. The values for all these metrics were enhanced, improving satisfaction and perception of care for the oncology patients. Working conditions of the healthcare providers were also improved.

There were several managerial key factors that allowed the successful development of this project in the singular Spanish NHS. The first key factor is the training courses received by the hospital staff, which were supported economically by the top management. These training courses involved oncology staff (e.g., oncologists and nurses) because initiatives are easier to be implemented when the staff involved in the process have helped to design the solutions. In addition, the implementation of the future state will be managed by hospital’s chief executive officer. The selection of the hospital process (e.g., ODH) was also critical because there was a lot of room for improvement and therefore the hospital staff, as well as the work team members, were highly involved. Keeping the project to a manageable size, continuously delimiting areas for improvement, and not intervening directly in the pharmacy laboratory, only establishing recommendations, was also key to the success of the project. Last but not least, it was also key not to interfere in the work of doctors within their consultations.

This paper demonstrated that VSM methodology helps healthcare professionals to deal with the ongoing challenge of improving patient care. It also contributed to healthcare theory and practice by applying, for the first time, lean methodologies to a Spanish ODH, whose peculiarities may accentuate resistance to change.

This study presents some limitations that are worth noting. The ODH considered is integrated into a large hospital and is not an independent institution. Due to this condition, the ODH shares resources with the rest of the hospital, making improvements more difficult to implement. Specifically, to make possible the ideal state, it would be necessary to level out all the workloads in the entire hospital. Because this change is not feasible in a reasonable time as it implies a global change in the entire hospital, other alternative future states are proposed. These solutions are not without limitations because patients need to go to the hospital 2 days instead of 1. However, patients were interviewed and they stated they would prefer this future state over the current situation because waiting times are still reduced. In addition, the future state 2 proposed requires a cost redistribution among the health institutions involved. This cost redistribution will be managed by the hospital’s chief executive officer and agreed with the regional government. On the other hand, being a health institution included in a health system where salaries are very rigid, resistance to change should not be forgotten. As has been mentioned, the training courses taken by the hospital staff are a key factor for improving commitment, but it is a limitation that must be taken into account.

The authors consider that the future work should go in the direction of working with the hospital managers to achieve the ideal future state, creating scheduling tools to coordinate the different departments involved in the studied ODH.

## Figures and Tables

**Table 1:** Metrics current state.

Metrics	Wait Time (Minutes)	Non-Value-Added Time (Minutes)	Value-Added Time (Minutes)			Length of Stay (Minutes)			VAR (Value-Added Time/Length of Stay)		
			Min	Med	Max	Min	Med	Max	Min	Med	Max
Current state	300	306	65	170	310	351	446	576	18.52%	38.12%	53.82%

**Table 2:** Final metrics.

Metrics	Wait Time (Minutes)	Non-Value-Added Time (Minutes)	Value-Added Time (Minutes)			Length of Stay (Minutes)			VAR (Value-Added Time/Length of Stay)		
			Min	Med	Max	Min	Med	Max	Min	Med	Max
Current state	300	306	65	170	310	351	446	576	18.52%	38.12%	53.82%
Ideal state	30	32	65	170	310	67	172	312	97.01%	98.84%	99.36%
Future state 1	80	86	65	170	310	131 (99 + 32)	226 (99 + 127)	356 (99 + 257)	49.62%	75.22%	87.08%
Future state 2	150	156	65	170	310	201 (32 + 169)	296 (32 + 264)	426 (32 + 394)	32.34%	57.43%	72.77%

## Data Availability

Submit requests to the corresponding author.
